# The Impact of Health Investment on Economic Growth: Evidence from China

**Published:** 2020-04

**Authors:** Qingyuan SHEN, Binbin CHANG, Guoyu YIN, Wendong WANG

**Affiliations:** 1. College of Information & Business, Zhongyuan University of Technology, Zhengzhou, China; 2. Pingdingshan Branch of Henan Rural Credit Union, Pingdingshan, China; 3. School of Law, Anqing Normal University, Anqing, China

**Keywords:** Governmental health investment, Residential health investment, Economic growth

## Abstract

**Background:**

Currently, China is carrying forward “Healthy China” construction. Thus, health investment has gradually become an important issue concerned by the Chinese government. Exploring the influence of health investment on economic growth under this background is of great theoretical and realistic significance for realizing economic transformation and upgrading in China.

**Methods:**

Thirty-one provincial regions in China were selected as research objects. Based on the panel data during 2000–2017, difference-generalized method of moment (D-GMM) and system-generalized method of moment (S-GMM) were comprehensively used to estimate the dynamic panel model from the national perspective, combining the fixed effects model (FE) estimation method to estimate the static panel model from the regional perspective, so as to investigate the relationships among governmental, residential health investment, and economic growth.

**Results:**

First, the governmental and residential health investments have positive effects on economic growth. Second, from the perspective of different regions, the governmental and residential health investments present positive correlations with economic growth, but the correlations present a progressively decreasing trend from the east to west.

**Conclusion:**

The Chinese government needs to steadily increase governmental health investment, elevate the level of residents’ health expenditure, promote the development of the health industry, and finally facilitate sustainable economic growth in China.

## Introduction

China is facing economic structural adjustment and transformation, gradual disappearance of population dividend, aggravated aging, and elevated economic downturn pressure. Consequently, health investment has become an important issue concerned by the Chinese government. The *Report on the Work of the Government in 2019* briefly stated that the Chinese government should continuously elevate health security levels, including basic old-age pension, basic medical care, and prevention and treatment of critical diseases, aiming to boost “Healthy China” construction (1). According to the *China Statistical Yearbook* (2) and *China Health Statistical Yearbook* (3), the health investment in China reached 70.952 billion RMB in 2000, accounting for 4.46% and 15.47% of the general budget expenditure and total health investment, respectively. In addition, the health investment of the Chinese government rose to 1,520.587 billion RMB in 2017, accounting for 7.49% and 28.91% of the general public budget expenditure and total health investment, respectively.

The health investment of the Chinese government increased yearly in 2000–2017 with an annular growth rate reaching 19.76%, and its proportion in public budget expenditure and total health investment both gradually increased. This indicates that the Chinese government is improving the health conditions of human capitals by adding health investment. The existing economic theories and practical development experience of developed countries manifest that human capital can exert an important promotional effect on economic growth. Human capital includes educational and healthy human capital (4). The educational human capital has a remarkable positive effect on economic growth is undisputed, however, whether accumulation of healthy human capital formed by health investment can become an endogenous power for economic growth as educational human capital has become a significant theoretical problem. In addition, investigating the relationship between health investment and economic growth is also an important realistic problem for facilitating the development of the massive health industry and realizing economic transition and upgrading in China.

Through literature review, most scholars affirmed the facilitating effect of governmental and residential health investments on economic growth (5–8). Some scholars believed that governmental health investment would form “crowding-out effect” on material capital and then obstructed economic growth (9, 10). Early-stage studies mainly concentrated on qualitative research (11, 12); however, recent studies began to include mathematical models to conduct quantitative research (13–16). Although the research achievements were abundant, the following problems still existed: First, the researching perspectives were too macroscopic.

Most scholars analyzed the relationship between health investment and economic growth from a national perspective. However, economic development levels in different regions of China differ considerably, and governmental health investment are not balanced. Thus, the research from a national perspective would cover the overall characteristics of China due to the offset of regional differences, making the statistics fail to truly reflect the relationship between actual health investment and economic growth in China. Second, even though governmental and residential health investments both belong to health investment, they have different effects on economic growth. The present literatures have overemphasized on the relationship between governmental health investment and economic growth with a lack of relationship between residential health investment and economic growth. Third, in terms of quantitative research methods, many scholars still relied on static panel and time-series ordinary least squares (OLS) models, and model variable selection might have a certain deviation. Therefore, these types of models could easily result in endogenous problem and then caused pseudo-regression problem.

On this basis, from the overall perspective of China and spatial perspectives of the eastern, western, central, and northeast regions of China, GMM was used in this study to handle endogenous problems to improve the model’s degree of fitting and reflect regional differences in governmental health investment, residential health investment, and economic growth in China.

This study can not only solve the deficiency regarding this aspect among mainstream literature in the field of health economics, but can also provide a new spatial perspective for scholars to investigate the effect of health investment on economic growth.

## Methods

### Modeling

Based on previous studies (14, 15), the extended Solow model, including health and education capital, was taken into consideration in this study. Its concrete functional form is as follows:

[1] $$Y=H^{\alpha }E^{\beta }K^{\gamma }{\left(AL\right)}^{1-\alpha -\beta -\gamma }$$

*Y* is the total output, *H* is the health capital input, *E* is investment in education human capital, *K* is material capital investment, *A* denotes comprehensive technical level, and *L* is labor input. *α*, *β*, *γ*, and 1 − *α* − *β* − *γ* are elasticity coefficient of health capital output, education capital output, material capital output, and technical labor output, respectively.

Total output *Y* refers to economic growth in this study. Empirical research objects are the effects of health investment on economic growth in 31 provinces of China during 2000–2017. Health investment includes not only public governmental investment but also private residential investment. Therefore, governmental and residential health investment can be introduced into Formula [1]. A group of control variables *X*, having an effect on economic growth are introduced into Formula [1] to improve the explanatory power of the model. In addition, natural logarithms are taken from two sides of Formula [1] in consideration of heteroskedasticity and collinearity problems existing in the model. Formula [1] can be extended into the following:

[2] $$InY_{\text{it}}=\alpha +\beta_{1}InHg_{\text{it}}+\beta_{2}InHp_{\text{it}}+\beta_{3}InE_{\text{it}}+\beta_{4}InK_{\text{it}}+\beta_{5}InL_{\text{it}}+\beta_{6}In{\text{X}}_{\text{i}}{}_{\text{t}}+{ɛ}_{\text{it}}$$

*Hg* is governmental health investment; *Hp* is residential health investment; *i* is province; *t* is year; and *ɛ_it_* is random disturbance term.

Given that important explanatory variables may be omitted in the model construction and that economic growth is a dynamic changing process, the influence of health investment on economic growth may have a certain hysteresis effect and long-term property. Therefore, the one period lagged variables of explained variables were used as explanatory variables and introduced into Formula [2] to establish a dynamic panel model:

[3] $$InY_{\text{it}}=\alpha +\beta_{0}InY_{\text{i},\;\text{t}-1}+\beta_{1}InHg_{\text{it}}+\beta_{2}InHp_{\text{it}}+\beta_{3}InE_{\text{it}}+\beta_{4}InK_{\text{it}}+\beta_{5}InL_{\text{it}}+\beta_{6}In{\text{X}}_{\text{i}}{}_{\text{t}}+{ɛ}_{\text{it}}$$

### Variable selection

The effect of health investment on economic growth is mainly investigated in this study. Thus, variables that can reflect economic growth are selected as explained variables, and the health investment variable is selected as the core explanatory variable. In addition, education human capital, which boosts economic growth; material capital, which is the material basis for economic development; and labor force, which is an indispensable constituent part of economic output of a country or region, also have important effects on economic growth; thus, they are called basic explanatory variables.

***1) Explained variable:*** Regarding variable selection for economic growth, most scholars’ select per capita gross domestic product (GDP), GDP, GDP growth rate, and per capita GDP growth rate as proxy variables for economic growth. In full consideration of the effect of population expansion on economic development and in reference to previous studies (14, 16), actual per capita GDP (*PCGDP_it_*), which is calculated through constant price in 2000, is finally selected as proxy variable for the explained variable, that is, economic growth.

***2) Core explanatory variable:*** Health investment includes governmental and residential health investment. Based on previous studies (17–21), the effect of population expansion is eliminated. Per capita governmental health investment (*PCGHI_it_*) and per capita personal health cash input (*PCPHI_it_*) are taken as proxy variables for core explanatory variables, that is, governmental and residential health investment, respectively.

***3) Basic explanatory variables:*** Based on previous studies (8, 22), per capita fixed investment (*PCMCI_it_*) is taken as proxy variable for material capital investment. The proportion of people having college degrees or above in the total population (*ED_it_*) is used as proxy variable for education human capital investment, and unemployment rate (*UE_it_*) is proxy variable for labor input.

***4) Control variables.*** Given that economic growth will be influenced by various factors, population aging, industrial structure, foreign trade, urbanization progress, and institutional change are taken as control variables for regression analysis based on previous studies (14, 17) to eliminate the endogenous problem of the model and improve the robustness of research conclusions.

*Population aging:* Population aging phenomenon is prominent in China at present. Health input triggered by population aging will certainly influence economic growth. Thus, the population aging problem should be considered to realize rational analysis of economic growth problem in China. Then, old-age dependency ratio (*ODR_it_*) is taken as proxy variable for population aging.

*Industrial structure:* Whether the industrial structure is reasonable or not directly decides economic development level. The proportion of non-agricultural output value in GDP (*IS_it_*) is selected as proxy variable for industrial structure.

*Foreign trade:* Foreign trade import and export are important factors that influence economic output. Thus, trade dependency (proportion of total volume of foreign trade in GDP: *TD_it_*) is taken as proxy variable for foreign trade.

*Urbanization progress:* The urbanization progress of a country or region will always influence economic activity density of this region and is closely related to economic output. Urbanization rate (*UR_it_*) is taken as proxy variable for urbanization progress.

*Institutional change:* The better the market economy system, the higher the proportion of output value of non-state-owned enterprises in industrial output, the better the business environment, and the higher the economic development degree. Non-nationalization rate (proportion of industrial output value of non-state-owned enterprises in gross output value of industrial enterprises above designated size: *NNR_it_*) is selected as proxy variable for institutional change.

According to the above variable selection, the dynamic panel model in Formula [3] is further concretized:

[4] $$\begin{array}{ll} lnPCGDP_{i,t} & =\alpha +\beta_{0}lnPCGDP_{i,t-1}+\beta_{1}lnPCGHI_{i,t}+\beta_{2}lnPCPHI_{i,t}+\beta_{3}lnPCMCI_{i,t}\\ & +\beta_{4}lnUE_{i,t}+\beta_{5}lnED_{i,t}+\beta 5lnX_{i,t}+U_{i}+{ɛ}_{i,t} \end{array}$$

*U_i_* is individual effect of each province.

### Data source

Basic data needed in empirical analysis of this study all comes from *China Statistical Yearbook*, *China Health Statistical Yearbook* and *China Industrial Statistical Yearbook* during 2001–2018. GDP price index, consumer price index, and investment price index in fixed assets are used for reduction processing of related variables with year 2000 taken as the base period to eliminate the effects of price fluctuation during 2000–2017 on variables. The descriptive statistics of variables are given as shown in Table 1.

**Table 1:** Descriptive statistical table of variable data of 31 provinces in China during 2000–2017

***Variable***	***Meaning***	***Mean value***	***Minimum value***	***Maximum value***
*PCGDP_it_*	Per capita GDP	30648.59	2759	128994
*PCGHI_it_*	Per capita governmental health investment	549.33	71.68	2695.55
*PCPHI_it_*	Per capita personal health investment	551.82	52.02	1710.6
*PCMCI_it_*	Per capita fixed investments	97814.44	718.96	1739559
*UE_it_*	Unemployment rate	0.04	0.01	0.07
*ED_it_*	Proportion of people having college degree or above in total population	0.01	0	0.48
*ODR_it_*	Old-age dependency ratio	0.12	0.06	0.22
*TD_it_*	Trade dependency	0.30	0.01	1.74
*NNR_it_*	Non-nationalization ratio	0.56	0.10	1.00
*UR_it_*	Urbanization rate	0.49	0.18	0.90
*IS_it_*	Proportion of non-agricultural output value in GDP	0.87	0.01	1.00

Table 1 shows that mean per capita GDP among 31 provinces in China during 2000–2017 was 30,648.59 RMB, presenting a gradual rising tendency. The province with the minimum and maximum per capita GDP was Guizhou Province in 2000 (2,759 RMB) and Beijing City in 2017 (128,994 RMB), respectively. Mean per capita governmental and per capita personal health investments were 549.33 RMB and 551.82 RMB, respectively. This result indicates that mean governmental health investment was smaller than mean residential health investment during 2000–2017.

## Results

### National analysis

D-GMM and S-GMM estimation methods were used to estimate dynamic panel data in 31 provinces in China during 2000–2017 (Table 2).

**Table 2:** Empirical estimation of the impact of health investment on economic growth from a national perspective

***Explanatory variable***	***China*** ***D-GMM***	***China*** ***S-GMM***
*PCGDP_i, t−1_*	0.886^***^ (43.75)	0.883^***^ (48.55)
*PCGHI_it_*	0.0809^*^ (1.93)	0.0715^*^ (1.95)
*PCPHI_it_*	0.0259^*^ (1.78)	0.0399^*^ (1.77)
*PCMCI_it_*	0.0337^*^ (1.89)	0.0422^**^ (2.27)
*UE_it_*	−0.101^**^ (−2.55)	−0.118^***^ (−2.90)
*ED_it_*	0.050^***^ (7.08)	0.0443 (1.33)
*ODR_it_*	−0.128^***^ (−6.70)	−0.0896^***^ (−5.45)
*TD_it_*	0.0628^***^ (13.50)	0.0268^***^ (7.02)
*NNR_it_*	0.0158 (1.02)	0.0130 (1.24)
*UR_it_*	0.171^***^ (7.51)	0.137^***^ (6.71)
*IS_it_*	0.0126^*^ (1.72)	0.0335 (1.19)
*Intercept*	1.838^***^ (8.30)	1.555^***^ (6.22)
*Autoregressive Process of order 1 test*	0.021	0.018
*Autoregressive Process of order 2 test*	0.603	0.853
*Sargan test P-value*	0.6611	0.7302
*Hansen test P -value*	1.0000	1.0000

Notes: Value in bracket represents t statistics.

^*, **^, and ^***^represent statistical significance at 10%, 5%, and 1%, respectively.

Table 2 shows that in D-GMM and S-GMM estimation methods, most explanatory variables pass the t test, indicating that most explanatory variables have significant effects on the explained variables. All *P*-values of Autoregressive Process of order 2 test in the model were greater than 0.1, manifesting that no remarkable second-order sequence correlation exists between residual sequence after difference processing and error term of S-GMM. The Sargan test *P*-value and Hansen test *P*-value were greater than 0.1, which means that the instrumental variables set by the model were exogenous and reasonable. Therefore, the estimation results of dynamic panel data of 31 provinces in China during 2000–2017 from D-GMM and S-GMM methods can explain the dynamic effect of China health investment on economic growth.

As shown in Table 2, influence coefficients of per capita GDP of lag 1 are 0.886 and 0.883 under D-GMM and S-GMM estimation methods, respectively. The influences are significant, which means that the current per capita GDP will be elevated by 0.886% and 0.883%, respectively, when each 1% increases per capita GDP of lag 1. The elasticity coefficients of the influences of per capita governmental and per capita personal health investment are both positive, where the former is slightly greater than the latter, and the influences are significant at 10% test level. For instance, every governmental and personal health investments under S-GMM estimation method are elevated by 1%, stimulating economic growth by 0.0715% and 0.0399%, respectively.

Furthermore, Table 2 shows that material investment, education human capital, urbanization rate, non-nationalization rate, foreign trade, and industrial structure all have positive promotional effects on economic growth, where the elasticity coefficient of the influence of material investment on economic growth is only 0.0337 under D-GMM estimation method, which is smaller than those of governmental and residential health investments, indicating that the positive effect of material capital investment on economic growth is weaker than contributions made by governmental and residential health investments on economic growth. The influence coefficient of education human capital on economic growth is low (0.05) under D-GMM estimation method, which is smaller than that of health investment. The elasticity coefficient of the influence of urbanization level on economic growth is high (0.171) under D-GMM estimation method, and the influence is significant.

The influence coefficients of non-nationalization rate on economic growth are 0.0158 and 0.0130 under the two estimation methods, and the influences are not significant. This result indicates that occupation rate of non-state-owned enterprises has a positive promotional effect, but this effect is not remarkable. Thus, further deepening state-owned structural reform, enhancing the market economy system, and enlarging the supporting force for private enterprises are necessary. The elasticity coefficient of the influence of foreign trade on economic growth under D-GMM estimation method is 0.171, and the influence is significant, manifesting that export still has a strong promotional effect on economic growth in China. The influence coefficients of proxy variable for industrial structure, namely, proportion of non-agricultural output in GDP, on economic growth are 0.0126 and 0.0335 under the two estimation methods, respectively.

The labor force’s proxy variable unemployment rate is negatively correlated with economic growth under the S-GMM estimation method and the coefficient of influence is −0.118, which is significant, indicating that each 1% increases in the unemployment rate will lead to a decrease of 0.118% per capita GDP and the unemployment rate will inhibit economic growth, which is consistent with theoretical expectations. The elasticity coefficient of the influence of proxy variable for aging, namely, old-age dependency ratio, on economic growth is −0.128 under D-GMM estimation method, and the influence is significant, which means that population aging has a negative correlation with economic growth in China.

### Regional analysis

Considering the great regional differences in economic development and health investment scale in China, China is divided in to four regions (eastern, western, central, and northeast regions) from a spatial view in this study, which aims to further verify the robustness of national analysis conclusions. In addition, in the analysis of different influences of health investments in the four major regions on their economic growth, data become dynamic long panel data (small n and large T), and the deviation of dynamic panel data is small, so generalized method of moment (GMM) is unsuitable. Thus, the within group estimator (fixed effect model, FE) is used to estimate the deviation. FE is used to measure the economic effect of health investment in different regions, and the estimation results are listed in Table 3. As shown in the table, Hausman test results are all smaller than 0.05, indicating that FE model is suitable for estimation. Table 3 shows that the influence coefficients of per capita governmental health investments in east, northeast, central, and west regions on per capita GDP are 0.296, 0.219, 0.174, and −0.0136, respectively.

**Table 3:** Regional panel data analysis results

***Explanatory variable***	***East (FE)***	***Center (FE)***	***Northeast (FE)***	***West (FE)***
*PCGHI_it_*	0.296^***^ (3.38)	0.174^**^ (2.16)	0.219^**^ (2.47)	−0.0136 (−0.14)
*PCPHI_it_*	0.126^*^ (1.38)	0.0892^*^ (1.36)	0.101^***^ (3.09)	0.0483 (0.48)
*PCMCI_it_*	0.0774^**^ (2.07)	0.420^***^ (8.76)	0.197^***^ (5.91)	0.256^***^ (6.12)
*UE_it_*	−0.419^***^ (−3.86)	−0.000994 (−0.01)	−0.00506 (−0.04)	−0.233^*^ (−1.86)
*ED_it_*	0.308^***^ (10.64)	0.172^**^ (3.03)	0.149^*^ (1.89)	0.0156 (0.96)
*ODR_it_*	−0.0715 (−0.69)	−0.128 (−1.07)	−0.379^***^ (−3.65)	0.190 (1.47)
*TD_it_*	−0.168^***^ (−2.76)	0.0587 (1.29)	−0.0452 (−1.00)	−0.0575^*^ (−2.30)
*NNR_it_*	0.273^***^ (2.64)	0.0913 (1.04)	−0.0376 (−0.36)	0.209^***^ (3.68)
*UR_it_*	1.598^***^ (9.46)	0.288 (1.03)	0.213 (0.98)	1.483^***^ (7.50)
*IS_it_*	0.0334 (1.42)	−0.947 (−1.61)	1.789^***^ (5.73)	3.227^***^ (7.17)
*Intercept*	8.645^***^ (18.36)	4.621^***^ (7.03)	3.186^***^ (4.02)	8.832^***^ (14.14)
R^*2*^	0.9269	0.9890	0.9934	0.9634
*Hausman test*	0.0000	0.0000	0.0003	0.0000

Notes: Value in bracket represents t statistics.

^*^, ^**^, and ^***^represent statistical significance at 10%, 5%, and 1%, respectively

Meanwhile, the influences in east, northeast, and central regions are all significant, and their governmental health investments have remarkable positive promotional effects on economic growth. However, the governmental health investment in the west region can inhibit economic growth, though not obviously. Overall, the influence of governmental health investment on economic growth presents a gradual progressive declining tendency from the east, northeast, center to the west. The influence coefficients of personal health investment on per capita GDP are 0.126, 0.101, 0.0892, and 0.0483, respectively.

## Discussion

According to the analysis of the results, there is a positive relationship between health investment and economic growth, and the problem raised in the introduction is resolved. The impact of health investment on economic growth in different regions of China is different.

The results in Table 2 indicate that economic growth of lag 1 can promote economic growth to a great extent, and China’s economic growth is of certain “inertia.” This finding is similar to the research conclusions drawn by most scholars and also accords with theoretical expectation. In addition, Table 2 shows that both governmental and residential health investments can boost economic growth to a certain degree, and government health investment has a slightly larger effect on economic growth. Residential health investment can stimulate economic growth through health consumption, but its effect is smaller than that of governmental health investment. Moreover, the positive effect of material capital investment on economic growth is already weaker than contributions of governmental and residential health investments to economic growth, which means that health investment has a certain crowding-out effect on material capital investment (23). Therefore, promoting the economic development purely by investing fixed asset and constructing infrastructure is already in a weak state, and it is better to get promoted by changing the economic development mode and adjusting the economic structure. The effect of education human capital on economic growth is also weaker than that of health investment because education investment scale in China has reached a high level in recent years (24). Moreover, the marginal effect brought by education human capital to economic growth has been gradually weaker than that of health human capital.

As shown in Table 3, governmental health investment has a remarkable promotional effect on economic growth in east, northeast, and central regions, presenting a gradual progressive declining tendency from the east, northeast to the center. This result indicates that in the region with higher degree of economic development, the positive effect of governmental health investment on economic growth becomes more obvious. The health investment in the western region has restrained the region’s economic growth. Because the western region’s fiscal revenue is low and it’s used less for health investment. Meanwhile, the governmental health investment may have a crowding-out effect on material capital investment, or the effect of government health investment on economic growth has not been apparent for a long time, but this inhibitory effect is temporary and not significant (25).

Hence, the west region should stick to the belief that health investment can promote economic development, continue to enlarge governmental health investment, and form “accumulative effect” of governmental health investment. In addition, the positive effect of residential health investment in the four regions on their economic growth also declines progressively from the east to west, manifesting that the region with developed economy has more vigorous personal health demand, and the effect of health investment on pulling economic growth is more evident.

## Conclusion

Based on the panel data of 31 provinces in China during 2000–2017, D-GMM and S-GMM estimation methods are used to comprehensively estimate the effect of health investment on the economic growth of China. The following conclusions are drawn: First, governmental and residential health investments can promote economic development to a certain degree. Second, when China is divided into four regions, the promotional effect of governmental and residential health investments on economic growth progressively declines from the east to the west, but the governmental health investment in the west can repress economic development, although not significantly.

## Ethical considerations

Ethical issues (Including plagiarism, Informed Consent, misconduct, data fabrication and/or falsification, double publication and/or submission, redundancy, etc.) have been completely observed by the authors.
